# Supplementary material for: Dietary lipids induce PPARd and BCL6 to repress macrophage IL-23 induction after intestinal injury and LPS exposure
Source: Sci Rep. 2025 Jul 27;15:27344. doi: 10.1038/s41598-025-12448-y (PMC12301443; doi:10.1038/s41598-025-12448-y)
Supplement: Supplementary file 1 — Supplementary Information. [file 41598_2025_12448_MOESM1_ESM.pdf]

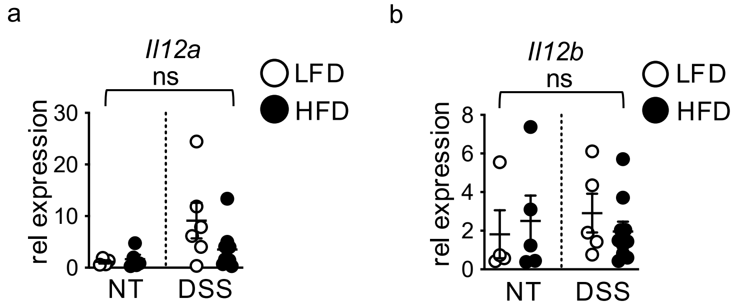

Supplementary Figure S1. Cecal gene expression in LFD and HFD non-treated and DSS mice. (**a**) *Il12a* (**b**) *Il12b*. Statistical comparisons were performed using One-way ANOVA with Bonferroni's multiple comparisons test.

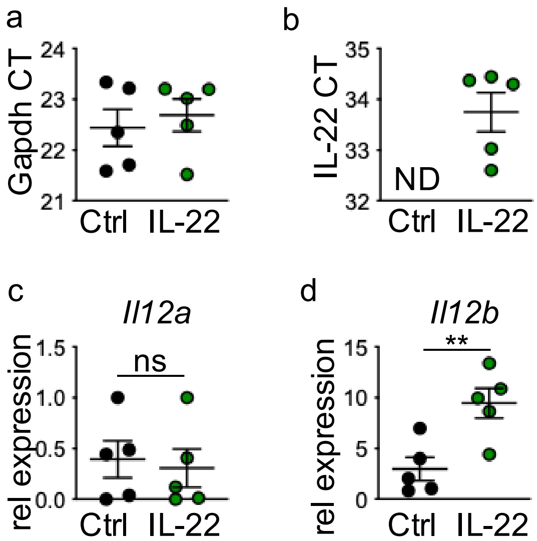

Supplementary Figure S2. Liver CT values at 1:10 dilution of (**a**) Gapdh and (**b**) IL-22 in mice with hydrodynamic delivery of a control or IL-22 expressing plasmid at day 9. Cecal gene expression of (**c**) *Il12a (p35)* (**d**) *Il12b (p40)* in HFD DSS mice with hydrodynamic delivery of control or IL-22 expressing plasmid. **P<0.01. Statistical comparisons were performed using Student's *t* test and if not indicated, a comparison is not significant.

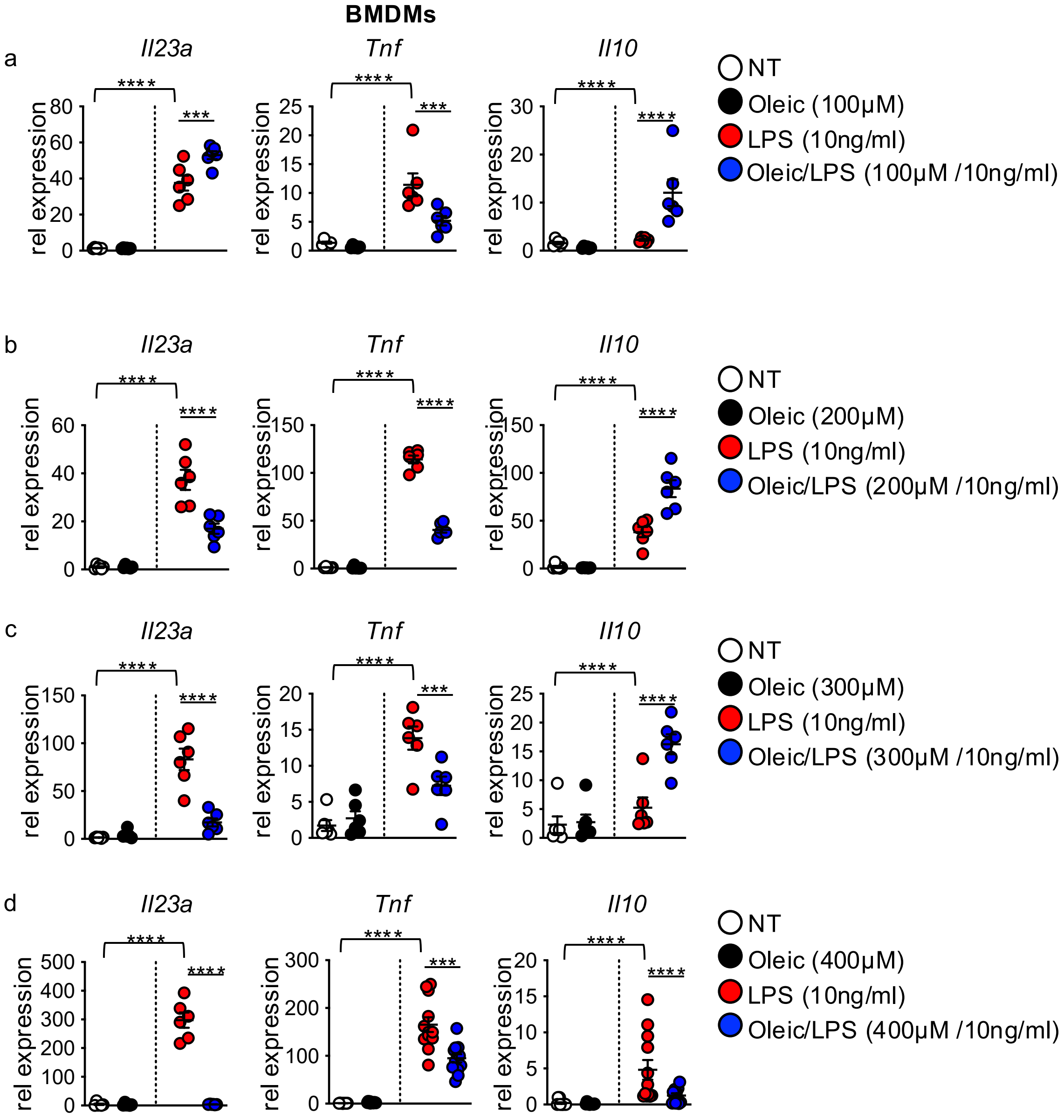

Supplementary Figure S3. Oleic acid dose-dependent regulation of macrophage LPS response. (**a-d**) *Il23a*, *Tnf*, and *Il10* gene expression in BMDMs left untreated or treated with oleic acid (100 - 400 µM) LPS (10ng), or oleic acid (100 - 400 µM) /LPS (10 ng) for 4 hours. Data are presented as mean ± SEM. ***P<.001, ****P<.0001. Statistical comparisons were performed using One-way ANOVA with multiple comparisons.

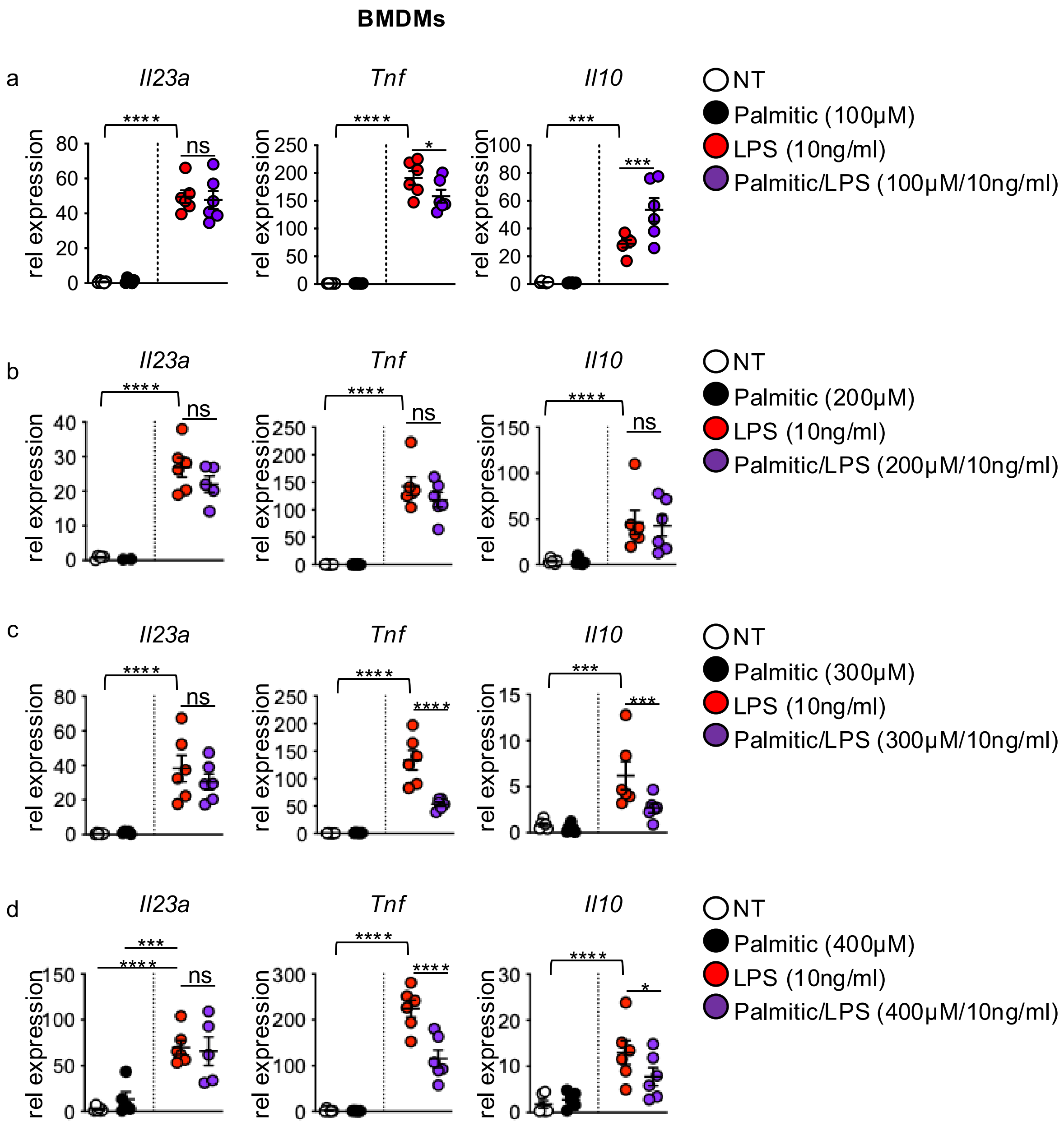

Supplementary Figure S4. Palmitic acid dose-dependent regulation of macrophage LPS response. (**a-d**) *Il23a*, *Tnf*, and *Il10* gene expression in BMDMs left untreated or treated with palmitic acid (100 - 400 μM) LPS (10ng), or palmitic acid (100 - 400 μM) /LPS (10 ng) for 4 hours. Data are presented as mean ± SEM. *P<0.05, ***P<.001, ****P<.0001. Statistical comparisons were performed using One-way ANOVA with multiple comparisons.

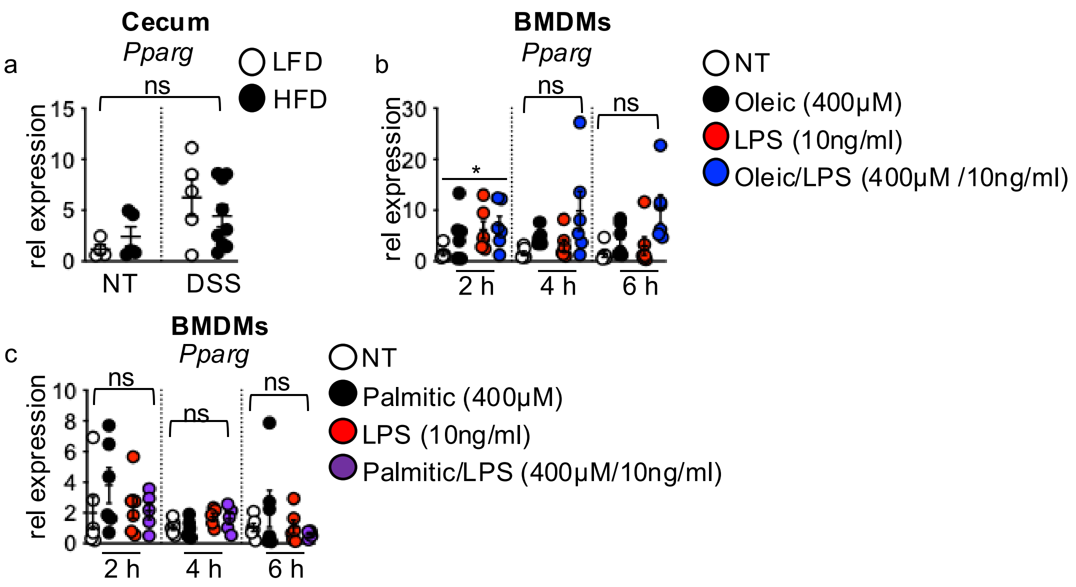

Supplementary Figure S5. Dietary lipid regulation of macrophage *Pparg* expression. (**a**) Cecal *Pparg* gene expression in LFD and HFD no-treated and DSS mice. *Pparg* gene expression in BMDMs left untreated or treated with (**b**) oleic acid (400 μM), LPS (10 ng), or oleic acid (400 μM) /LPS (10 ng) or (**c**) palmitic acid (400 μM), LPS (10 ng), or palmitic (400 μm) /LPS (10 ng) for 4 h. *P<0.05. Data are presented as mean ± SEM. Statistical comparisons were performed using One-way ANOVA with multiple comparisons.

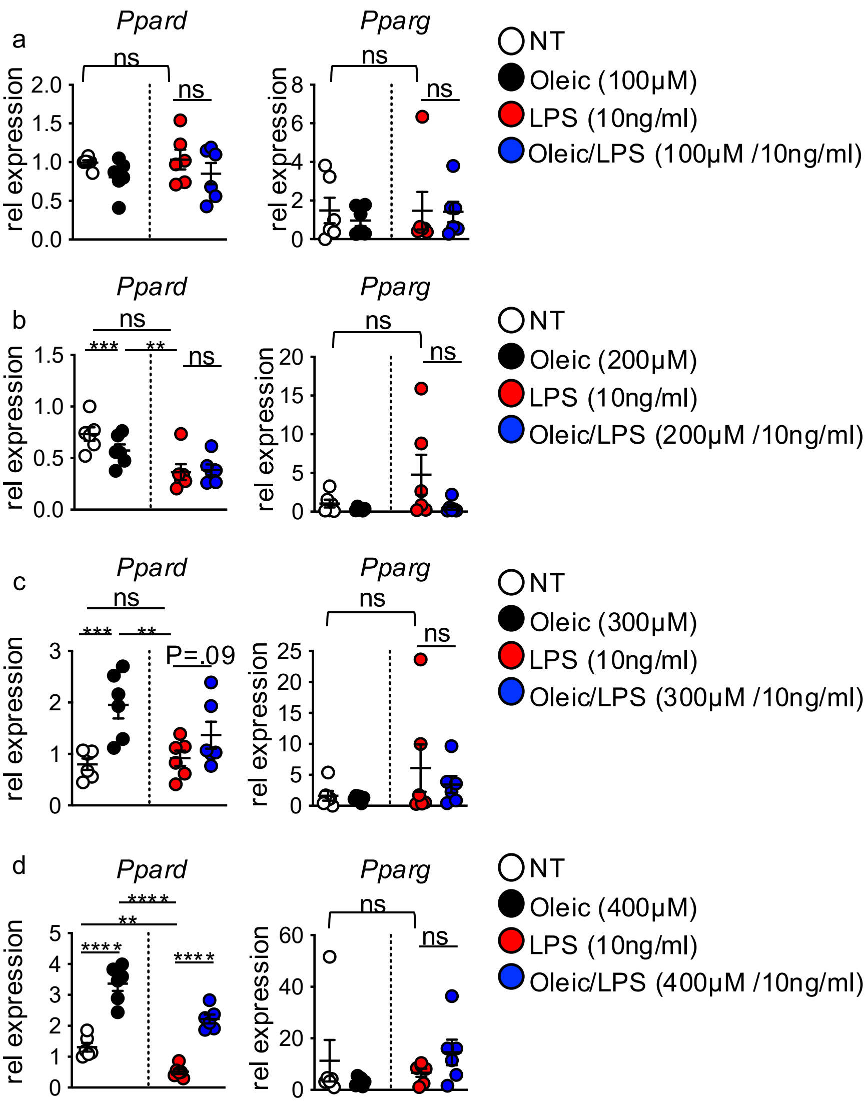

Supplementary Figure S6. Oleic acid dose-dependent regulation of macrophage *Ppard* and *Pparg* expression. (**a-d**) *Ppard* and *Pparg* gene expression in BMDMs left untreated or treated with oleic acid (100 - 400 µM), LPS (10ng), or oleic acid (100 - 400 µM) /LPS (10 ng) for 4 hours. Data are presented as mean ± SEM. **P<0.01, ***P<.001, ****P<.0001. Statistical comparisons were performed using One-way ANOVA with multiple comparisons.

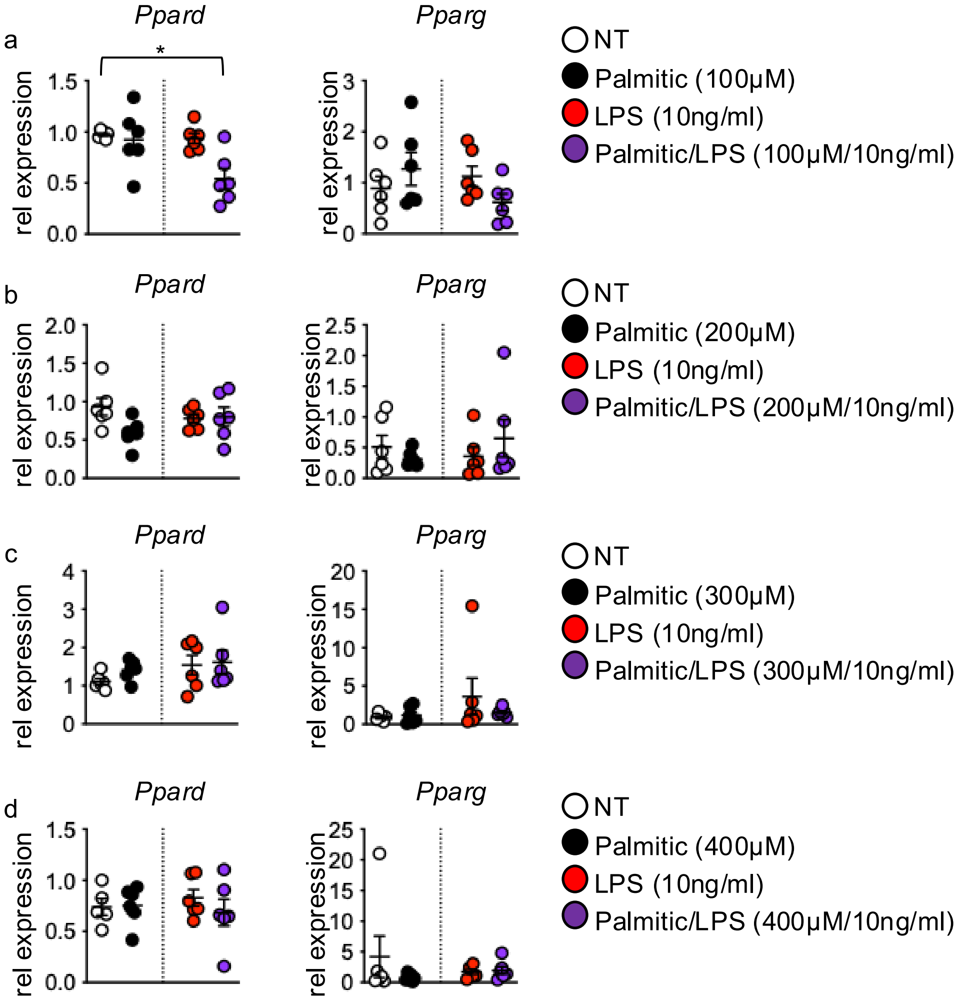

Supplementary Figure S7. Palmitic acid dose-dependent regulation of macrophage *Ppard* and *Pparg* expression. (**a-d**) *Ppard* and *Pparg* gene expression in BMDMs left untreated or treated with palmitic acid (100 - 400 µM), LPS (10ng), or palmitic (100 - 400 µM) /LPS (10 ng) for 4 hours. Data are presented as mean ± SEM. **P<0.01, ***P<.001, ****P<.0001. Statistical comparisons were performed using One-way ANOVA with multiple comparisons.
